# Parents’ experiences of communication with neonatal intensive-care unit staff: an interview study

**DOI:** 10.1186/s12887-014-0304-5

**Published:** 2014-12-10

**Authors:** Helena Wigert, Michaela Dellenmark Blom, Kristina Bry

**Affiliations:** Institute of Health and Care Sciences, The Sahlgrenska Academy at University of Gothenburg, Box 457, Gothenburg, SE 405 30 Sweden; Division of Neonatology, Sahlgrenska University Hospital, Gothenburg, 416 85 Sweden; Department of Pediatric Surgery, Sahlgrenska University Hospital, Gothenburg, 416 85 Sweden; Department of Pediatrics, The Sahlgrenska Academy at University of Gothenburg, Gothenburg, 416 85 Sweden

**Keywords:** Communication, Hermeneutic lifeworld approach, Neonatal intensive care, Parental experience

## Abstract

**Background:**

An infant’s admission to a neonatal intensive-care unit (NICU) inevitably causes the parents emotional stress. Communication between parents and NICU staff is an essential part of the support offered to the parents and can reduce their emotional stress. The aim of this study was to describe parents’ experiences of communication with NICU staff.

**Methods:**

A hermeneutic lifeworld interview study was performed with 18 families whose children were treated in the level III NICU at a university hospital in Sweden. The interviews were analysed to gain an interpretation of the phenomenon of how parents in the NICU experienced their communication with the staff, in order to find new ways to understand their experience.

**Results:**

Parents’ experience of communication with the staff during their infant’s stay at the NICU can be described by the main theme ‘being given attention or ignored in their emotional situation’. The main theme derives from three themes; (1) *meeting a fellow human being*, (2) *being included or excluded as a parent* and (3) *bearing unwanted responsibility*.

**Conclusions:**

This study shows that parents experienced communication with the NICU staff as essential to their management of their situation. Attentive communication gives the parents relief in their trying circumstances. In contrast, lack of communication contributes to feelings of loneliness, abandonment and unwanted responsibility, which adds to the burden of an already difficult situation. The level of communication in meetings with staff can have a decisive influence on parents’ experiences of the NICU.

The staff should thus be reminded of their unique position to help parents handle their emotional difficulties. The organization should facilitate opportunities for good communication between parents and staff through training, staffing and the physical health care environment.

## Background

The admission of an infant to a neonatal intensive care unit (NICU) inevitably causes emotional stress for the parents and hence complicates the parent–infant bonding process [1-5]. The parents are vulnerable during the infant’s hospitalization [4,6,7]. Communication between parents and NICU staff is an essential part of the support offered to the parents and can reduce their emotional stress [8-11]. To better meet the communication needs of parents, it is important to know how they experience communication with the staff at the NICU.

Communication as a concept is defined as the conveying or sharing of information between people [12]. Information is provided in *what* is said verbally and *how* the message is communicated non-verbally [13]. Communication in this study means succeeding in conveying both information and emotional support to the parents while being responsive to their needs.

Previous studies have shown that parents of infants hospitalized in a NICU felt helped by information about their child’s state of health and treatment, and by opportunities to discuss their experiences with staff members [11,14-16]. Parents are dependent on staff for the care of their child and for help to cope with their experiences [2,17]. In order to manage uncertainty about the child’s health, parents need to receive factual information, as well as support and engagement from health care staff [15]. An empathetic attitude on the part of doctors and nurses seems to make a difference to parents’ experience of communication in the NICU and to their relations with staff members [11]. Family-centered care (FCC), which is a way of caring for children and their families, can be practiced in NICU. The relationship between parents and staff is the core in FCC which necessitates an open and honest communication between the parents and nursing staff [18].

Previous studies have shown that the NICU staff do not always meet parents’ needs and may not always experience communication problems the same way as parents do [10,19-21]. Research that considers parents’ experience of all staff members and uses a qualitative, open-ended method to gain data on a deeper level is sparse. The aim of this study was to describe parents’ experiences of communication with NICU staff.

## Methods

### Study design

The study was conducted using the hermeneutic lifeworld approach, as described by Dahlberg, Dahlberg and Nyström [22]. The lifeworld is defined as the everyday world in which we live our lives and take all our activities for granted. Hermeneutic philosophy highlights the idea that being in the world and interpreting it is the basis of understanding, and that language is an essential tool because it gives us access to other people’s experiences [23]. Hermeneutic lifeworld research requires the researchers to have an open and sensitive attitude to the phenomenon they are studying. An open and sensitive attitude is described in terms of “bridling pre-understanding” which involves a willingness to listen, see and understand deeper meanings of the phenomena through a distancing and reflective attitude to unfamiliar experiences [22]. The researchers must therefore try to find new ways of seeing, interpreting and understanding phenomenona [23].

### Setting

Parents were recruited from a level III NICU [24] at a university hospital in Sweden that provides care for approximately 1,000 newborns annually. The NICU has 22 beds divided among two intensive care and two intermediate care rooms, and a staff of 120, including doctors, registered nurses and nursing assistants. The NICU has a high turnover of patients who are transferred to a level II neonatal unit or other paediatric care unit, or discharged home, once their medical condition is sufficiently stable. The NICU has a family-centred care policy [18] and parents are welcome to spend as much time as they want in the unit with their child.

### Ethics

Ethical approval was obtained from the Regional Research Ethics Committee in Gothenburg, Sweden, registration number 535–10. All parents gave written informed consent and were informed about guaranteed confidentiality and the right to discontinue the interview at any time.

### Participants

Using medical records, we first identified families who met the following inclusion criteria: (a) neonatal care was initially given in a level III NICU, (b) less than 12 months had elapsed since discharge from the NICU and (c) the parent spoke and understood Swedish. In this type of qualitative study there are usually 15–20 respondents and variability among respondents is important for achieving reliable data [22]. The aim was therefore to ensure sample variation with respect to infant sex, gestational age at birth, birth weight and length of stay in the NICU. A purposive sample of 18 families was therefore selected. We contacted the families by telephone and all of them agreed to participate in the study. They were permitted to decide which of the parents would participate, as well as the time and place of the interview.

Twenty-seven parents (11 fathers and 16 mothers) in 18 families were interviewed within the first year of their child’s life (mean 5.6 months). Altogether 19 interviews were held. For eight families, the two parents were interviewed together whereas one-parent interviews were conducted for the other nine families. For one family, both parents were interviewed separately. Five interviewees were first-time parents and 22 were parents for the second time. Three parents were of non-Scandinavian descent. The mothers’ age ranged from 26 to 44 years (mean 33 years) and the fathers were aged from 26 to 41 years (mean 34 years). Four families had twins. Seventeen infants were born prematurely, of whom seven were born extremely prematurely (under 28 gestational weeks at birth). Five infants were born at full term. The 22 infants stayed in the NICU for 11 to 120 days (mean 46 days, median 33 days). Eighteen infants suffered from respiratory distress to a varying degree, eight suffered from cerebral haemorrhage or neonatal stroke, and three were born with a congenital anomaly. All infants were given intravenous drugs during their NICU stay, 13 had mechanical ventilation, 13 had nasal continuous positive airway pressure (CPAP) and six had surgery.

### Interviews

Open-ended interviews were conducted and recorded digitally in the parent’s home. Interviews lasted between 23 and 70 minutes. All interviews began by asking the parents to provide a narrative of their experiences of communication with the staff at the NICU, with a question formulation such as ‘Please tell me about your experiences of communication with the staff when your child was treated in the NICU’. All parents were encouraged to speak openly about their experiences, and follow-up questions were used to confirm the researchers’ understanding of the narratives provided. Since the last interviews revealed essentially no new data, no additional families were contacted.

### Analysis of the interviews

The interviews were transcribed verbatim and the analysis was based on principles described by Dahlberg et al. [22]. It is important in this hermeneutic lifeworld approach not to use any predetermined hypotheses or any theories. Like all forms of text analysis, the interpretative analysis is a dialogue with the message of the text. All the text was read without preconceived ideas and critically several times to understand parents’ experiences of communication with the NICU staff, including underlying meanings and explanations that were not immediately obvious. The meanings in the text were condensed, compared and grouped in clusters, which were compared and contrasted. The analytic phase was thus open and flexible with a distancing, reflective and critical approach. The interpretations of the parts of each transcript were constantly compared with the interpretation of the whole transcript, in order to decide whether there was a discrepancy between the understanding of the parts and the understanding of the whole [22,23]. Three interpretative themes of the parents’ experiences of communication with NICU staff were identified and finally integrated into a main interpretation in order to understand further meanings of the phenomenon”.

## Results

The parents’ experience of communication with the staff when their infant was treated in the NICU can be described by the main theme ‘being given attention or ignored in their emotional situation’. The main theme derives from three themes: (1) *meeting a fellow human being*, (2) *being included or excluded as a parent* and (3) *bearing unwanted responsibility*.

### Meeting a fellow human being

The parents described their distress over their child’s medical condition and appreciated it when the doctors and nurses paid attention to their situation through their communication. The parents felt supported when they were met with compassion, as when the doctor in the conversation showed her feelings. It was comforting to meet the human being behind the professional role.

“*The doctor listened, the doctor was also a person … she showed that she was also a fellow human being in the whole thing; she said,* ‘*but God, here I am, saying horrible things to you, but of course I have to say what I say now’.*” (Father)

The parents felt they were taken notice of when the staff responded to their need for information by listening attentively and calmly answering their questions. Unhurried conversation was reassuring and gave parents the opportunity for emotional relief. Parents also appreciated occasions when staff conveyed sensitivity to their need for consolation. The staff gave parents space to be alone, but also offered to share their burden. The parents did not have to communicate with words how they felt, but rather the staff could sense their state of mind and were there in the background when the parents needed them.

“*We noticed that they were keeping an eye on the situation … They were hanging around, they were there and started talking a bit and could tell if you wanted to talk*.” (Mother)

The parents felt secure with the staff they regularly communicated with and had thus created a relationship with. Having a designated doctor and nurse contact in the NICU for their child provided continuity and felt important to the parents. Getting to know the staff created an atmosphere of trust in which parents dared to talk about their needs and wishes.

“*We had our contact nurses … it felt really nice because we could come to them with these extra requests*.” (Mother)

The parents felt that conversations with staff created the opportunity for a break from a reality that was difficult to live with. During small talk with the nurse on the ward they got the opportunity to be more than the parent of a sick child; they got to be the person they were before the child was born. Humour in their communication with the staff could defuse the situation at the NICU and make it less painful. Laughing with the staff gave them strength to cope with circumstances.

“*Communicating, talking about other things, being allowed to forget reality for a while … there is so much focus on the child. Sometimes it’s like you have no life outside*.” (Mother)

### Being included or excluded as a parent

The parents felt invited to communicate when the staff took the time to explain the child’s care and treatment to them and invited them to participate in the child’s care. This encouragement to care for the child strengthened parental bonding with the child; parents stated that they had received ‘parent training’ that made them confident in their own ability to care for their baby after they were discharged from the NICU. Through communication an inclusive parenting with mutually trusting cooperation between both parties could arise, which strengthened the parent’s identity as a parent.

“*There is a communication together with us, [they] answer questions, provide support, tell us what we can do and what they will help with*.” (Father)

The parents felt that they were dependent on communication with the staff to get information about their child and to get support from the staff to participate in their child’s care. When parents were not given information about their child’s care and treatment, they felt themselves excluded in their parenting. For example, not being allowed to participate in the ward round involving their child to hear some of the information that emerged was described as being deprived of their parental role.

“*It was weird, because it was my child who was lying there, so I wanted to know what they said; if it had been me who was sick, I would have been allowed to hear it; now there was not really anyone who could speak for him… I was afraid that I was only getting the information that they wanted to talk about at that time*.” (Mother)

The parents explained that they got the most information from the staff at the beginning of the child’s hospitalization but at that time it could be difficult to take in information because the mother was most often still recovering from the birth. As time went by, the amount of information and the number of discussions, mainly with doctors, declined after the child’s condition stabilized.

“*It would have felt good to have a review discussion there, what happened after the birth … because I have no idea of what happened there, I know that I’ve thought about that afterwards*.” (Mother)

The parents stated that they were often left waiting for some time for information about their child’s illness. When the answer was uncertain, or conversations with the doctor were postponed or information failed to materialize, the parents suffered. They were filled with worry and perceived themselves at the mercy of their imagination with unanswered questions such as ‘What is wrong with my child?’ and ‘What are they doing to my child?’

“*We sat in the room furthest away, in the private side room, sat there all day and no one even came in to see us … it was several days before I even found out what was wrong with my child … nobody told us*.” (Mother)

The parents described communicative situations in which they felt lonely. They felt abandoned when one of the staff members gave them bad news about their child’s condition in passing. It was hard to take in messages from the staff when no one stayed with them to discuss what the news meant for their child.

“*Then there was this doctor who just came in for a few minutes, really stressed out, and burst out with, ‘Yes, these three brain injuries and this one at the back are of course very dangerous and blah, blah, blah’ and then he went out. We were completely devastated and just cried and wondered, ‘Were you talking about our child, has he got another brain injury?’*” (Mother)

Communication with staff could leave an emotional impression on the parents, such as when they received negative information about their child’s illness – information that was painful to receive and hard to bear. The parents stated that they had difficulty understanding what was being said and that it was about them and their child. Those memories preoccupied them, even after the hospital stay in the NICU.

“*When you as a lay person hear the term cerebral infarction, you freeze, you don’t understand that it’s happening to you, this can’t happen to us … There was one doctor, he came unannounced to our room and then you realize that there was some imminent danger, something the matter that wasn’t as it should be. A doctor never comes unannounced, not with positive news anyway. … It took us about a day to regain our composure, so to speak*.” (Mother)

Parents who experienced a lack of trust in staff sometimes chose not to communicate their distress. They did not want to show how hurt they were, but instead they put on a brave face, which created feelings of abandonment.

“*I have not told this to anyone because whenever I talk about that time, I say we’ve been treated very well … but now that we are discussing communication and staff, I can’t hide it*.” (Mother)

### Bearing unwanted responsibility

The parents felt that, in their communication with the staff, they adapted to each member of staff’s personality and their availability for conversation. They learned the different responsibilities of the various professionals and what roles they had in communicating with parents. For example, spontaneous and urgent discussions with the doctor were often associated with negative information about the child’s condition, whereas the nurse usually brought good news to them directly.

“*The longer it took before we got to talk to a doctor, the better the result. Compared with how often they, the parents of the child next ours got to talk to the doctor, we realized that our son was very healthy. … The nurse was often the one to bring positive news straight away.*” (Mother)

Even the structure of the conversation differed between the different professions. Conversations with nurses often took the form of emotional support whereas conversations with doctors focused primarily on information about the child’s medical condition and treatment. It could be difficult for parents to understand the doctor’s information during the conversation, in which case the parents had to take the initiative to ask the nurse for an explanation of what had been said. The parents felt that they had to take an unwanted responsibility upon themselves for successful communication with the nursing staff, when they wanted this to be the responsibility of the staff instead. They also had to act as messengers and inform the staff at the maternity ward about their child’s health care needs. Similar situations occurred when the child was transferred to another unit and the parents had to brief the staff there.

“*Communication between the maternity ward and Neonatal could be improved. They had failed to schedule the hearing test. They didn’t know if it was the maternity ward or Neonatal that booked it, so I had to check it myself. It was several weeks after we had arrived home.... Then I got worried that there might be more things they had missed.*” (Mother)

Likewise, they felt that the staff had unspoken expectations of them as a parent: how much they were expected to be present with their child, what they should participate in and take responsibility for in the care of the child. This could make the parent feel insecure in their parenting role. When they felt that the staff were not communicating with them about their child’s care and treatment, they had to request this information themselves, which was difficult when the parent did not know what to ask about.

“*Vague communication, should we remember when the child needs feeding or should the staff do it, and sometimes in case we forgot … it was as if we were supposed to take on the responsibility.*

### Main interpretation

A main interpretation emerges from the three themes of the parents’ experiences of communication with NICU staff. Their experience can be understood as *being paid attention to or ignored in their emotional situation*. Parenthood in the NICU begins as an involuntary journey whose ultimate goal is a well-functioning family. The parents go through their time in the NICU either in communication with the staff or in the absence of such communication. The parent is dependent on communication with the staff, and attentive communication exists when the staff member gives full attention and is responsive to the parent’s situation; this means that parents feel that they are being listened to in meetings with the staff. Attentive conversations with the staff create a trusting relationship that gives parents peace of mind and the ability to orient themselves in their chaotic situation.

Where communication is absent, parents feel isolated in their situation, which amplifies their concerns about their child and leads to a sense of abandonment. The parents will then be forced to take responsibility for their situation and make efforts to establish communication with the staff.

## Discussion

The main theme that emerged was that parents in the NICU experience communication with staff as ‘being given attention or ignored in their emotional situation’. The main theme derives from three themes: *meeting a fellow human being*, *being included or excluded as a parent*, and *bearing unwanted responsibility*.

The parents in the study felt that they were given attention in their situation when the staff made themselves available and showed compassion, for example by expressing their own feelings in words, in their communication with the parent. This finding is in line with previous studies that demonstrated the importance of emotionally supportive communication if parents are to experience good communication with the NICU staff [11,14,16]. Weiss, Goldlust and Vaucher [16] reported that the availability of conversations is a key factor in parents’ perception of health care staff as empathetic [16].

The ability to understand another person’s situation is based on the feeling of empathy [25-28], which means being emotionally responsive to the other person’s needs without judging or criticizing them. Responding with empathy and compassion makes health care meaningful, but may require energy beyond the professional role of health care staff [29]. In a recently published study by Turner et al. [10] investigating nurses’ perspectives on emotional support to parents in the NICU, it emerged that both lack of senior staff and understaffing in general added to the burden of a busy and emotionally intense environment; this is an environment in which neonates have severe and life-threatening illnesses and parents are grieving [10]. In common with previous studies, our study underlines the necessity of advanced training for staff and the minimization of work-related obstacles to support the role of the neonatal nurse in providing emotional support [10,11,30,31]. A study by Boss, Hutton, Donohue and Arnold [32] found that trainee neonatologists were taught technical skills and medical knowledge, but wanted more training in communication with parents of seriously ill children.

Parents in this study were either encouraged to communicate with staff or excluded from communication, which included or excluded them in their parenting. Previous studies have shown that conversations between parents and staff diminish as the child’s condition is stabilized [8,33], but our study is the first to show that this clearly contributes to feelings of abandonment, according to the parents’ narratives.

As in other studies [21,33], our study demonstrated that encouragement from the staff to talk to them was important for giving parents a sense of their own significance for their child.

In a study by Younger [34], suffering is described as bringing with it loneliness or alienation from others and a feeling of heartbreak [34]. Being the parent of a child cared for in the NICU can be described as a situation involving suffering and where health care staff with good communication skills shows compassion. Compassion allows the staff to be affected by the other’s experience [35]. This study found that parents felt that there was a human being behind the profession when the staff showed themselves to be touched by the parent’s plight. Health care based on compassion means providing fellowship by sharing the person’s experiences [26,36] and thus trying to alleviate their suffering [35,36]. To be able to see and respond to this suffering, there must be a communication between the individual and the health care staff, and the staff member must ‘*see with the eye of the heart*’ [37]. Martinsen [37] uses the biblical story of the Good Samaritan to illustrate the human need for compassion in a difficult situation, in this case, the parent’s vulnerable situation of having a child cared for in the NICU. Having the patient’s suffering in mind is reflected in the staff member’s behaviour towards the patient: seeing, listening to and giving full attention to the patient in this situation. The staff member’s conduct has the power to reduce or worsen the patient’s suffering [37]. Håkonsen-Martinsen [38] argued that Martinsen’s health care philosophy is relevant in both nursing and clinical medicine. By ‘seeing with the eye of the heart’, the staff member can be moved by the patient’s situation – in this study, the parent’s situation in the NICU – which makes it easier for the staff member to communicate with and gain the trust of the patient. A study by Fenwick, Barclay and Schmied [39] found that mothers more easily develop a trust in nurses if they can chat with each other on a personal level about things beyond the hospital world [39]. In our study it emerged that parents of children in the NICU experienced a brief respite as they chatted with the staff about things that had nothing to do with their situation in the NICU. From this perspective, the communication between parents and staff provides fellowship, which can help make it easier for parents to bear their experiences in the NICU.

FCC, as practiced in the NICU in this study, emphasizes the importance of open and honest communication between parents and staff [18]. The findings of this study show that there is a gap between what is considered to be important, on the one hand and what was actually practiced and how the parents in in this study experienced their communication with staff, on the other hand. The parents felt that communication with staff meant being in the hands of other people; they were dependent on the staff and adapted themselves to their terms, such as being forced to take responsibility for communication themselves. Several studies have previously shown that parents in the NICU experience powerlessness and handle the situation by seeking to participate in the care of their child [1,5,11,40]. Studies concerning parents’ participation in communication regarding decisions about their child’s care and treatment at the NICU often frame this in the context of ethics [17,38,41]. Fegran, Helseth and Slettebø [17] maintained that nurses have a special ethical responsibility because, in a very vulnerable emotional situation for the parents, they have the power to decide how much involvement parents should have in their child’s care. Alderson, Hawthorne and Killen [19] likewise argued that parents’ participation in decision-making concerning their child’s care is an important part of good communication. They referred to this as the many minor choices and decisions offered to parents in the daily care of their child, which can involve major responsibilities and activities for them as parents [19].

People create relationships with one another through communication and, when the relationship between patient and staff is central [37], which was also demonstrated between parents and staff in this study, it is interesting to reflect on the significance of the staff members’ personal qualities. Martinsen [37] maintained that health professionals can choose how they relate to the patient: either by considering their own feelings and therefore believing they know what is best for a patient in those circumstances or, as Martinsen [37] advocated, becoming ‘involved’ in the patient, and considering that person’s situation. It is our hope that staff at the NICU will become ‘involved’ in the parent’s situation and thus they and the parent will be able to meet in an existential communication. It is hard for the parents of a child cared for in the NICU to cope with their situation but, as this study shows, the situation can be eased through attentive communication.

It should be noted that our study was conducted at a single NICU (level III) in Sweden, where both the health insurance system and the organization of healthcare delivery promote the presence of both parents during the infant’s hospital stay. The study context and the small number of participants may thus limit the applicability of the findings to other settings. Nonetheless, the variations in parent and infant characteristics support context transferability and thus strengthen the applicability of the results [22].

## Conclusions

This study shows that parents in the NICU experience communication with staff to be essential for them to manage their situation in the unit. Attentive communication offers the opportunity for a respite from reality, for compassion and relief. A lack of communication contributes to feelings of loneliness and being abandoned, as well as unwanted responsibility, which adds to the burden of an already difficult situation. The level of communication in meetings with the staff can have a decisive influence on parents’ experiences in the NICU. The staff should be reminded and remain aware of their unique position to help parents process emotional difficulties and therefore through communication share the parents’ situation, respond to their emotions and encourage conversation. The organization should also facilitate opportunities for good communication between parents and staff through training, staffing and the physical healthcare environment.

## Abbreviations

NICU: Neonatal intensive-care unit
